# Intramuscular endometriosis of the forearm: a case report

**DOI:** 10.1007/s00256-024-04648-w

**Published:** 2024-03-26

**Authors:** Kira L. Smith, Lulu He, Julie Adhya, Lisa Ercolano

**Affiliations:** 1 Drexel University College of Medicine, 60 N. 36th Street, Philadelphia, PA 19104 USA; 2 Department of Diagnostic Imaging and Radiology Allegheny Health Network, Allegheny General Hospital, 320 East North Avenue, Pittsburgh, PA 15212 USA; 3 Department of Orthopaedic Surgery Allegheny Health Network, Allegheny General Hospital, 320 East North Avenue, Pittsburgh, PA 15212 USA

**Keywords:** Intramuscular endometriosis, Forearm, Skeletal radiology, Orthopaedic oncology

## Abstract

Endometriosis is a disorder that commonly affects females of reproductive age and is defined as the presence of endometrial glands or stroma outside the uterine cavity. Patients typically present with cyclical pain during menses. Endometriosis can be characterized as endopelvic or extrapelvic depending on the sites involved. We report a case of a 40-year-old, right-hand-dominant, female who presented with a painful mass in her right proximal forearm. She was ultimately diagnosed with intramuscular endometriosis and underwent surgical excision.

## Introduction

Endometriosis is a common gynecologic condition characterized by the presence of endometrial tissue in sites other than the uterus. Endometriosis can be either endopelvic or extrapelvic based on the area of the endometrial tissue implantation. Typical locations for endopelvic endometriosis include the ovary, pelvic peritoneum, vagino-rectum diaphragm, and uterosacral ligament [1–3]. Extrapelvic endometriosis is rare and has a lower incidence, accounting for approximately 12% of endometriosis cases [4]. In theory, endometriosis can occur in all organs of the body, including the gastrointestinal tract, urinary tract, respiratory tract, and musculoskeletal system [5–7]. Intramuscular endometriosis has been reported in the trunk muscles, pelvic muscles, and extremities. Symptoms can be highly variable, but patients may complain of cyclical pain during menstruation, a palpable mass, or swelling that increases at time of menstruation.

Diagnosis of intramuscular endometriosis is difficult and requires a high index of suspicion. It is best managed by an interprofessional team approach to achieve a prompt diagnosis and optimize patient outcomes. Once the proper diagnosis is made, treatment in the form of hormonal or surgical management can be considered. This case report describes clinical and radiological findings as well as treatment modalities of endometriosis that appeared in the proximal forearm of a 40-year-old patient.

## Case report

A 40-year-old, right-hand-dominant, female (G3P3003) presented to the Orthopedic Department with an 18-month history of an intermittently painful mass in the medial aspect of her right forearm. The mass would become increasingly tender and firm in the days before menses and during menstruation. Her past medical history was notable for relapsing remitting multiple sclerosis (MS), hypertension, dyslipidemia, anxiety, and morbid obesity. She denied any excessively heavy or painful menses.

**Fig. 1:**
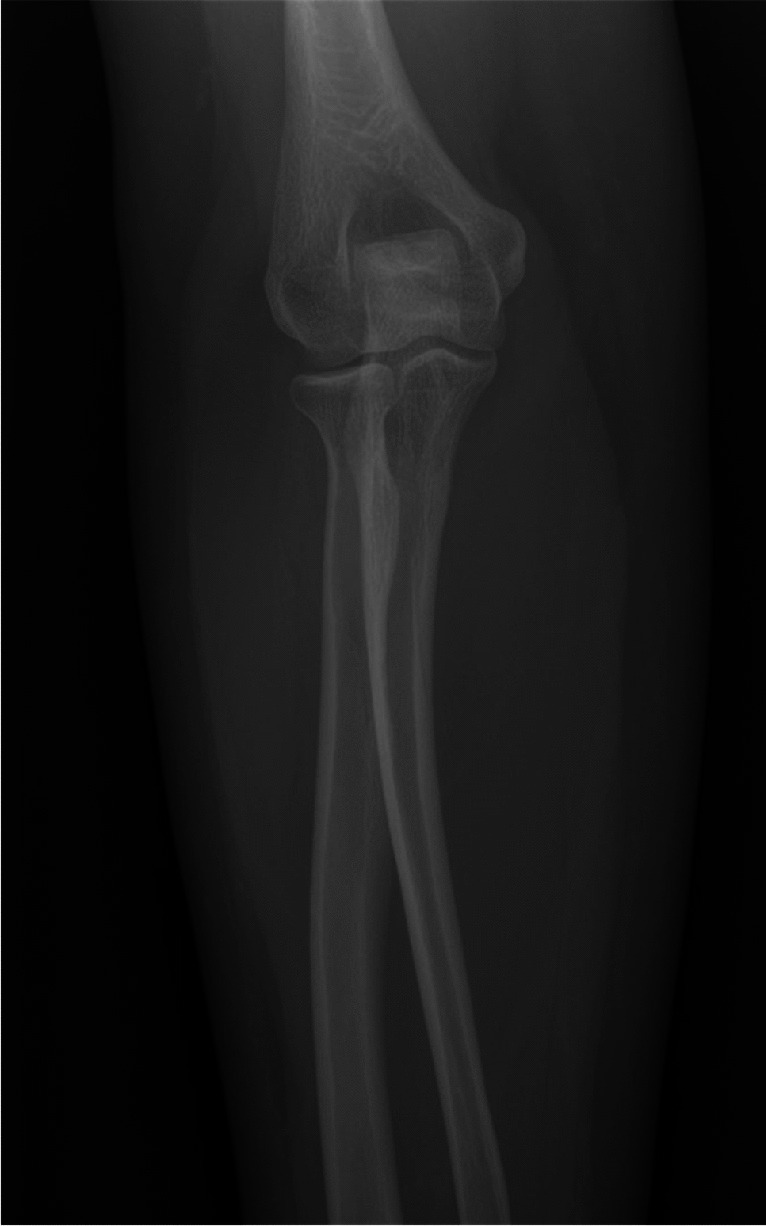
AP radiograph of the right forearm demonstrates no radiographic osseous or soft tissue abnormality

**Fig. 2:**
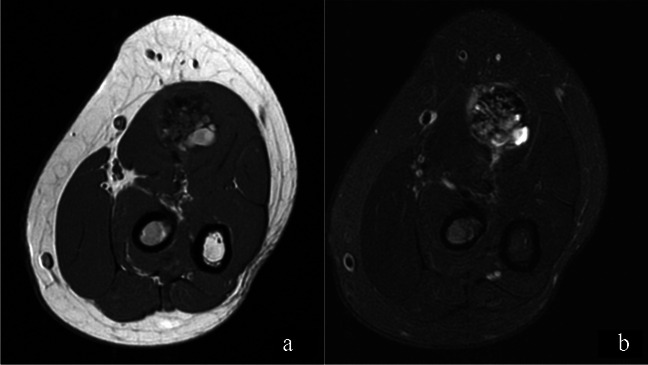
Axial T1W (**a**) and T2W (**b**) images of the forearm demonstrates a mixed signal intensity mass in the pronator teres muscle belly. This lesion demonstrates areas of intrinsic T1 signal hyperintensity and is predominantly T2 hyperintense. There are small cystic areas within the lesion and there is mild perilesional edema

**Fig. 3:**
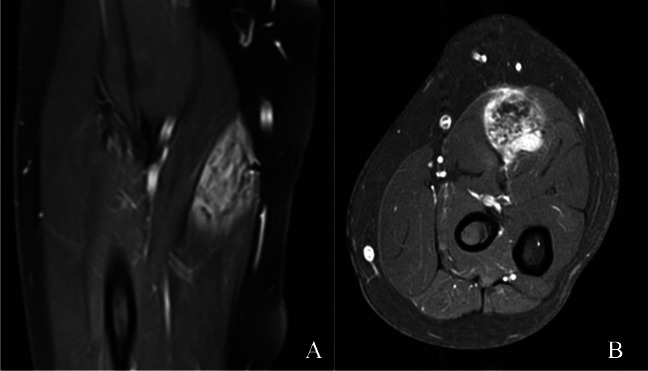
Coronal (**A**) and axial (**B**) post contrast T1W images demonstrates avid enhancement

**Fig. 4:**
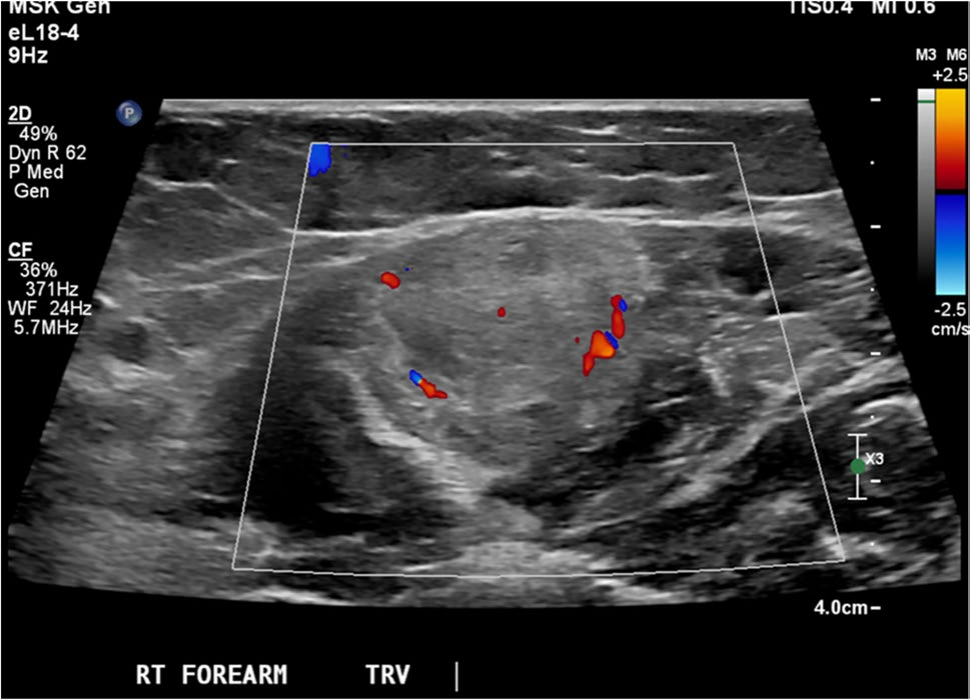
Ultrasound images demonstrate a heterogenous oval mass that is hyperechoic to adjacent musculature. There is internal color doppler flow

**Fig. 5:**
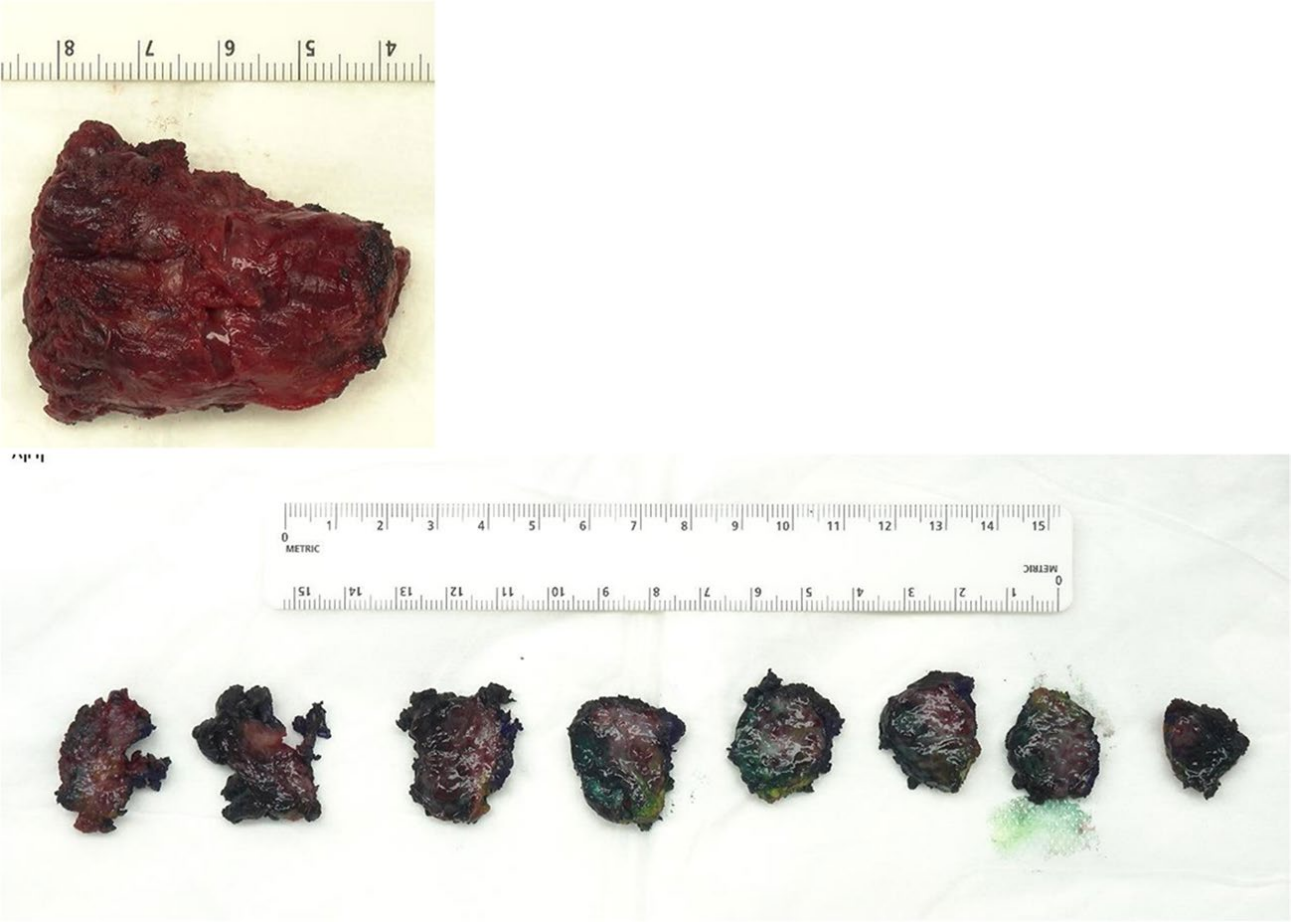
Gross specimen – 5-cm solid soft tissue mass

**Fig. 6:**
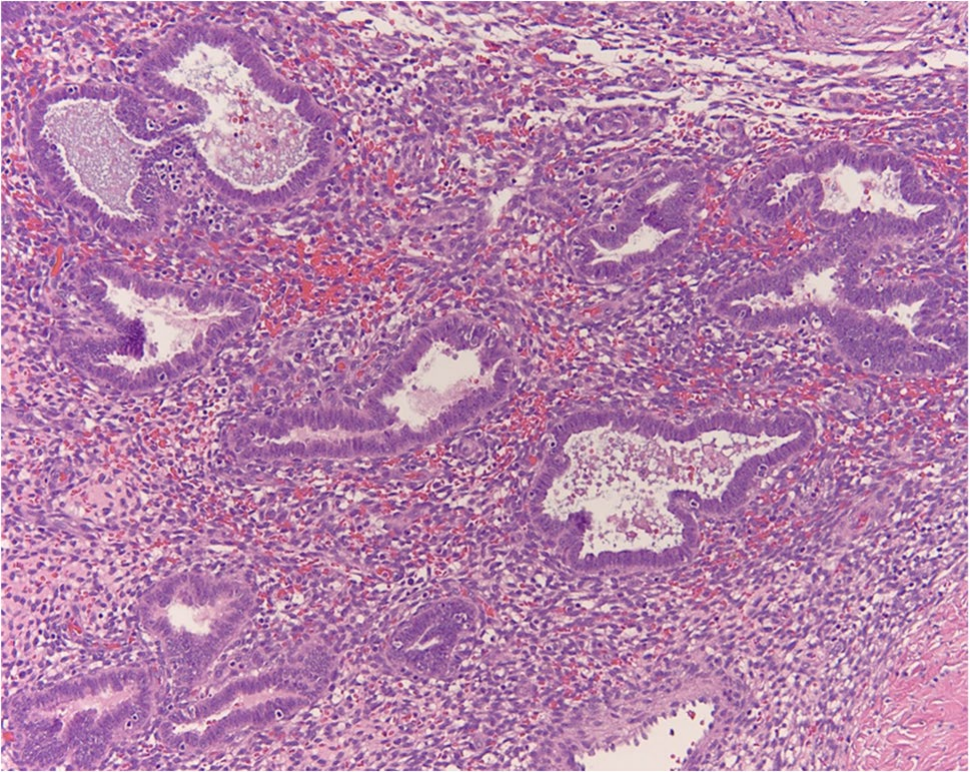
Endometrioid-type glands with hyperchromatic, elongated nuclei. Surrounding the glands are endometrial-type stromal cells and hemorrhage which all together are consistent with features of endometriosis

**Fig. 7:**
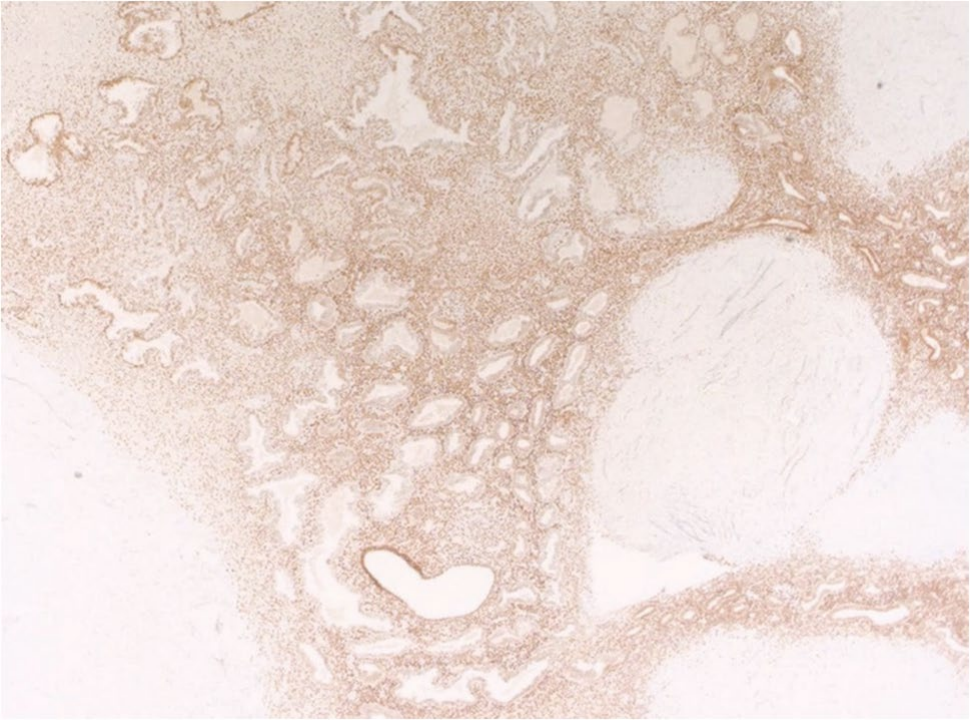
The endometrial glands and endometrial stroma are highlighted by the estrogen receptor immunohistochemical stain

Physical examination revealed a small palpable mass in the right flexor pronator musculature and was tender to palpation. Previous imaging, including radiographs of the right forearm demonstrated no acute osseous or soft tissue abnormality (Fig. 1). Magnetic resonance imaging (MRI) without intravenous contrast showed an oval intramuscular mass in the anterior compartment of the proximal forearm, measuring approximately 2.2 × 2.1 × 3.0 cm. The mass demonstrated heterogenous T2 signal and cystic changes (Figs. 2, 3). There was no invasion of deeper musculature or fascia. The decision was made to proceed with ultrasound-guided core needle biopsy of the lesion. On ultrasound imaging, the mass was circumscribed and hypoechoic. The mass also demonstrated internal doppler flow (Fig. 4). Unfortunately, the core needle biopsy was nondiagnostic. Therefore, the patient elected to undergo open biopsy with frozen section diagnosis, which was consistent with endometriosis (Figs. 5, 6, 7).

The patient was referred to Obstetrics/Gynecology for further evaluation and to discuss treatment options. Additional history obtained at that time revealed that she had delivered all three of her children by uncomplicated Cesarean section. Physical examination did not result in any concern for pelvic endometriosis. Dienogest, a progestin-based medication, was recommended with the goal of suppressing the proliferation of the endometrium to decrease symptoms. As the patient was still working to achieve remission of her MS with medications, she did not wish to add another medication at that time and declined the progestin medication. Approximately 18 months after initial presentation to the orthopedic oncology clinic, the patient’s symptoms worsened with pain now radiating into the elbow, distal forearm, and hand. She elected to trial Dienogest; however after several months, this did not improve her symptoms. After discussion with the orthopedic surgeon, the patient ultimately decided to undergo surgical excision of the mass. At 6 months postoperatively, the patient remains significantly improved compared to pre-resection. She does not very mild soreness to the forearm at the time of menstruation but states it does not bother her.

## Discussion

Extrapelvic endometriosis can occur at almost any site in the musculoskeletal system. The patient presented in this case report had intramuscular endometriosis to the proximal forearm. To our knowledge, this is the first known case of an intramuscular lesion to this location published in the English literature. Previous reports of intramuscular endometriosis to the upper extremity are limited, with one case to the trapezius muscle and two cases to the deltoid muscle [8–10]. More common intramuscular locations include the abdominal muscles, pelvic floor muscles, hip muscles, and lower limb muscles [4].

There are several theories to account for the origin of endometriosis including retrograde menstruation, coelomic metaplasia, local trauma, lymphatic and vascular metastasis, or stem cells. Retrograde menstruation refers to the reflux of menstrual debris containing viable endometrial cells through the fallopian tubes into the peritoneal cavity [1–3]. This theory is widely accepted but does not explain cases in which lesions are found outside of the peritoneal cavity. Coelomic metaplasia is the transformation of undifferentiated coelomic epithelial cells in extrauterine locations into glandular endometrial tissue [1–3]. However, musculoskeletal nerve tissue has a different embryological origin from germ cells of the pelvic peritoneum, so this does not explain intramuscular endometriosis. Canis et al performed a systematic review, suggesting that local traumatic events may trigger endometriosis [11]. The relationship between trauma and endometriotic lesions is strongly suggested by the very low recurrence rate observed in most studies after a complete excision [12]. In the setting of musculoskeletal endometriosis, the initial trauma may have been unnoticed by the patient or forgotten by the time the disease is diagnosed [11, 13].

Lymphatic and vascular metastasis is the transport of endometrial cells or stem cells through lymphatic and blood vessels and has been proposed as an origin of extrapelvic endometriosis. This theory is supported by previous pelvic surgery being cited as a risk factor for development of endometriosis of the skeletal muscular system [4]. The stem cell origin posits that stem cells of endometrial origin may enter the angiolymphatic space passively during menstruation and enter the circulation system to find environmentally friendly “soil” for seeding [2]. The strength of the endometrial stem cell theory is that it not only fits the retrograde menstruation model but also explains the pathogenesis of endometriosis outside the abdominal cavity.

Despite extensive research on the pathogenesis of endometriosis, there is no single theory that explains all the different clinical presentations and pathological features in endometriosis. Our patient had a history of three previous Cesarean sections and therefore it is presumed that her intramuscular endometriosis arose from lymphatic and vascular metastasis. However, it cannot be confirmed whether the transported cells were endometrial cells or stem cells.

The diagnosis of extrapelvic endometriosis is challenging as clinical presentation varies and imaging manifestations may be confusing. Ye et al systematic review of skeletal muscular system endometriosis revealed that only 53.6% of patients have local pain and 23.2% of patient’s symptoms were irrelevant to their menstrual cycle [4]. Furthermore, only 44.4% of patients that underwent fine-needle aspiration (FNA) with ultrasound or CT monitoring were confirmed to have endometriosis by FNA tissue pathology [4]. The relatively low success rate of FNA may be a limitation in prompt diagnosis. MRI with and without intravenous contrast is considered the modality of choice for evaluation of intramuscular soft tissue masses. Extrapelvic endometriosis have been described as containing areas of T1 hyperintensity due to methemoglobin [13]. On T2-weighted imaging, endometriomas can demonstrate high signal intensity; however, high iron concentrations can result in low-signal intensity appearance, a feature reflecting cyclic hemorrhage [13]. Due to cyclic degeneration and proliferation of endometrial tissue, MRI appearance can vary depending on lesion age, the presence of cystic change, and associated blood products.

The therapeutic options in the treatment of endometriosis depend on the extent of the disease, the patient’s needs, and the desire to maintain the reproductive capacity. These options include simple observation, medical treatment, surgical treatment, and combined therapy. An important consideration in the treatment of intramuscular endometriosis is the presence or absence of endopelvic endometriosis. Ye et al reported that 47.4% of patients with skeletal muscular system endometriosis did not have concurrent endopelvic endometriosis [4]. In the absence of endopelvic endometriosis, if the patient is asymptomatic and the lesion is not impacting any surrounding structures simple observation may be reasonable.

Medical treatment is aimed at improvement of pain and size reduction of the endometrioma. Progestin based contraceptive pills are one of the main drug treatments and work by altering the hypothalamic-pituitary-gonadal axis, which subsequently suppresses ovulation [7]. Additionally, GnRH agonists can be used and work by blocking the production of ovarian-stimulating hormones, lowering estrogen levels, and preventing ovulation [7]. Surgical resection of lesions is the main treatment when medical treatment is ineffective. Careful surgical technique is imperative in intramuscular endometriosis, as tissue implantation at the site of incision can lead to scar endometriosis [14]. Our patient did pursue observation for approximately 18 months as she focused on solidifying her MS medication regimen. Her symptoms progressively worsened and were unresponsive to medical treatment, leading to the decision to surgically excise the lesion.

Histologic appearance of endometrial tissue undergoes physiologic and morphologic changes throughout the menstrual cycle. During the menstrual phase, estrogen and progestin levels fall resulting in breakdown of endometrial stroma with the presence of inflammatory cells and blood [15]. The proliferative phase is driven by increasing estrogen levels and is characterized by numerous mitotic figures in glands and stroma [15]. The secretory phase is driven by progestin and is represented by irregularly shaped glands with a single layer of columnar or cuboidal cells [15]. Biopsy of our patient’s lesion demonstrated endometrioid-type glands with hyperchromatic, elongated nuclei, consistent with features of endometriosis. Immunostaining with estrogen receptor (ER) and paired box gene (PAX8) were positive, further supporting the diagnosis of endometriosis. Although ER concentrations are lower in endometriotic tissue when compared with normal endometrium, its presence is still helpful in distinguishing between glandular tissues [16]. PAX8 is a highly sensitive epithelial marker for extragenital endometriosis [17].

In summary, we report an extremely rare case of intramuscular endometriosis in the proximal forearm. Extremity endometriosis may be included in the differential diagnosis of soft tissue tumors when symptoms of pain or a palpable mass occur in the extremities of women of reproductive age, particularly those masses with a cyclic pattern, which coincides with the menstrual cycle.

## References

[1] Taylor HS, Kotlyar AM, Flores VA. Endometriosis is a chronic systemic disease: clinical challenges and novel innovations. Lancet. 2021;397(10276):839–52. 10.1016/S0140-6736(21)00389-5. 33640070 10.1016/S0140-6736(21)00389-5

[2] Wang Y, Nicholes K, Shih IM. The origin and pathogenesis of endometriosis. Annu Rev Pathol. 2020;15:71–95. 10.1146/annurev-pathmechdis-012419-032654. 31479615 10.1146/annurev-pathmechdis-012419-032654 PMC7980953

[3] Zondervan KT, Becker CM, Missmer SA. Endometriosis. N Engl J Med. 2020;382(13):1244–56. 10.1056/NEJMra1810764. 32212520 10.1056/NEJMra1810764

[4] Ye H, Shen C, Quan Q, Xi M, Li L. Endometriosis of the skeletal muscular system (ESMS): a systematic review. BMC Womens Health. 2023;23(1):37. Published 2023 Jan 26. 10.1186/s12905-023-02184-8. 36703173 10.1186/s12905-023-02184-8 PMC9878923

[5] Andres MP, Arcoverde FVL, Souza CCC, Fernandes LFC, Abrão MS, Kho RM. Extrapelvic endometriosis: a systematic review. J Minim Invasive Gynecol. 2020;27(2):373–89. 10.1016/j.jmig.2019.10.004. 31618674 10.1016/j.jmig.2019.10.004

[6] Davis AC, Goldberg JM. Extrapelvic endometriosis. Semin Reprod Med. 2017;35(1):98–101. 10.1055/s-0036-1597122. 27992931 10.1055/s-0036-1597122

[7] Machairiotis N, Stylianaki A, Dryllis G, et al. Extrapelvic endometriosis: a rare entity or an under diagnosed condition? Diagn Pathol. 2013;8:194. Published 2013 Dec 2. 10.1186/1746-1596-8-194. 24294950 10.1186/1746-1596-8-194 PMC3942279

[8] Gennari L, Luciani L. Un caso di endometriosi del muscolo trapezio [A case of endometriosis of the trapezius muscle]. Tumori. 1965;51(5):361–5. 10.1177/030089166505100506. 5863426 10.1177/030089166505100506

[9] Kaur J, Arora A, Gaba S, Rastogi P, Bagga R. Tracing the journey of endometrium, from womb to arm: deltoid endometriosis. J Obstet Gynaecol India. 2020;70(6):529–32. 10.1007/s13224-019-01292-6. 33417641 10.1007/s13224-019-01292-6 PMC7758389

[10] Nagamoto Y, Hashimoto N, Kakunaga S, et al. Endometriosis in the deltoid muscle: a case report. Eur J Orthop Surg Traumatol. 2012;22:497–500. 10.1007/s00590-011-0851-5.

[11] Canis M, Bourdel N, Houlle C, Gremeau AS, Botchorishvili R, Matsuzaki S. Trauma and endometriosis. A review. May we explain surgical phenotypes and natural history of the disease? J Gynecol Obstet Hum Reprod. 2017;46(3):219–27. 10.1016/j.jogoh.2016.12.008. 28403918 10.1016/j.jogoh.2016.12.008

[12] Horton JD, Dezee KJ, Ahnfeldt EP, Wagner M. Abdominal wall endometriosis: a surgeon’s perspective and review of 445 cases. Am J Surg. 2008;196(2):207–12. 10.1016/j.amjsurg.2007.07.035. 18513698 10.1016/j.amjsurg.2007.07.035

[13] Basu PA, Kesani AK, Stacy GS, Peabody TD. Endometriosis of the vastus lateralis muscle. Skeletal Radiol. 2006;35(8):595–8. 10.1007/s00256-005-0052-6. 16308716 10.1007/s00256-005-0052-6

[14] Botha AJ, Halliday AE, Flanagan JP. Endometriosis in gluteus muscle with surgical implantation. A case report. Acta Orthop Scand. 1991;62(5):497–9. 10.3109/17453679108996657. 1950501 10.3109/17453679108996657

[15] Deligdisch L. Hormonal pathology of the endometrium. Mod Pathol. 2000;13(3):285–94. 10.1038/modpathol.3880050. 10757339 10.1038/modpathol.3880050

[16] Nisolle M, Casanas-Roux F, Wyns C, de Menten Y, Mathieu PE, Donnez J. Immunohistochemical analysis of estrogen and progesterone receptors in endometrium and peritoneal endometriosis: a new quantitative method. Fertil Steril. 1994;62(4):751–9. 10.1016/s0015-0282(16)57000-9. 7523199 10.1016/s0015-0282(16)57000-9

[17] Arakawa T, Fukuda S, Hirata T, et al. PAX8: A Highly sensitive marker for the glands in extragenital endometriosis [published online ahead of print, 2019 Feb 14]. Reprod Sci. 2019:1933719119828095. 10.1177/1933719119828095. 10.1177/1933719119828095 30764713

